# Concurrent working memory task increases or decreases the flanker-related N2 amplitude

**DOI:** 10.3389/fpsyg.2022.962153

**Published:** 2022-09-02

**Authors:** Hua Wei, Yuan Yao, Lili Zhou

**Affiliations:** ^1^Department of Psychology, Suzhou University of Science and Technology, Suzhou, China; ^2^School of Psychology, Shanghai University of Sport, Shanghai, China

**Keywords:** working memory load, selective attention, flanker, ERPs, N2 amplitude

## Abstract

Concurrent working memory (WM) task reduces available attentional control resources to perform the flanker task. However, controversy exists as to whether concurrent WM task increases or decreases flanker-related N2 amplitude. In a flanker task experiment, individuals were confronted with a low, middle, or high WM load task, while electroencephalography (EEG) data were recorded. The ERP results showed a larger flanker-related N2 amplitude while completing a middle or high WM load task compared to a low one. However, completing an additional high WM load task could not increase flanker-related N2 amplitude versus completing an additional middle WM load task. In sum, these results suggest that WM load can impair top-down cognitive control processes, thereby hampering flanker task performance. Importantly, the present study supports the account of flanker-related N2 processes linked to top-down attentional control resource allocation, but challenges the account of flanker-related N2 reflecting response conflict processes.

## Introduction

Individuals often struggle to handle multiple tasks simultaneously. According to Lavie’s load theory of selective attention, increased working memory (WM) load impairs top-down attentional control processes, resulting in greater distractor interference (Lavie et al., 2004). In a dual-task paradigm, individuals were asked to perform a flanker task while simultaneously completing a WM task of either low or high load (Lavie et al., 2004; Lavie, 2005, 2010). The process of flanker task concerns the individuals’ ability to monitor and resolve the conflict using the attentional control resources. Concurrent WM task depletes the limited attentional control resources, and then lead to the available attentional control resources are limited to complete the flanker task. Consequently, an additional high WM load may increase the reaction times (RTs) for response conflict trials, leading to an increased interference effect (Lavie et al., 2004; Qi et al., 2014; Zhang and Luck, 2015; Murphy et al., 2016; Wei and Zhou, 2020).

Scalp recorded event-related potentials (ERPs) can offer real-time, temporal resolution of neural responses of conflict detection, monitoring, and resolution. In previous ERP studies, concurrent WM tasks increased the interference effect, which was presented as increased N2 amplitude and reduced P3 amplitude for response conflict trials in the flanker task (Pratt et al., 2011; Qi et al., 2014; Wei and Zhou, 2020). In the flanker task, the ERP component N2 is a fronto-central stimulus-locked component with a latency between 200 and 400 ms, which exhibits the following pattern: it is larger in experimental conditions providing incongruent response options than in experimental conditions providing congruent response options (Kopp et al., 1996; Folstein and Van Petten, 2008; Wei et al., 2021). Presently, the flanker-related N2 wave is thought to reflect one of two different and opposite cognitive processes: (1) response conflict processes, and (2) top-down attentional control resource allocation (Folstein and Van Petten, 2008; Tillman and Wiens, 2011; Qi et al., 2014; Wei et al., 2021).

The first account posits that flanker-related N2 is often considered an important indicator of response conflict (Yeung et al., 2004; Folstein and Van Petten, 2008; Yeung and Nieuwenhuis, 2009; Clayson and Larson, 2011; Larson et al., 2014; Qi et al., 2014). Flanker-related N2 is associated with the activation of the anterior cingulate cortex (ACC), which may signal conflict; the conflict signal in turn triggers strategic accomplishment of a given task, implemented by the prefrontal areas (Carter et al., 1998; Van Veen and Carter, 2002a,b; Groom and Cragg, 2015). The N2 and RTs reflect the same cognitive processes: response conflict processes; larger N2 amplitudes are always related to longer RTs for correct trials, especially for incongruent trials (Yeung et al., 2004; Yeung and Nieuwenhuis, 2009; Clayson and Larson, 2011). According to this point, increased N2 amplitude and RTs for incongruent trials (compared to congruent trials) reflect greater response conflict in incongruent trials (versus congruent trials).

In recent years, the second account has begun to emerge as a new perspective; it posits that flanker-related N2 may reflect top-down attentional control processes used to focus on the task-relevant aspects of a situation (Bartholow et al., 2005; Tillman and Wiens, 2011; Wei et al., 2021). N2 and RTs demonstrate different cognitive processes. According to this assertion, the increased N2 amplitude for incongruent trials (compared to congruent trials) indicates that individuals use more top-down attentional control resources to complete incongruent trials than congruent trials. Tillman and Wiens (2011) found a larger flanker RTs interference in the condition of 20% (versus 80%) of incongruent trials, but a larger flanker N2 amplitude interference in the condition of 80% (versus 20%) of incongruent trials. This is because a lower incongruent trial frequency is associated with greater response conflict while completing the incongruent trials. Thus, the results showed a larger flanker N2 amplitude interference in the condition of 80% (versus 20%) of incongruent trials, which disagrees with the first account but supports the second one. If the flanker-related N2 reflects the response conflict processes, the change in N2 amplitude interference should be similar than the change in RTs interference. Thus, Tillman and Wiens (2011) proposed that flanker-related N2 may reflect top-down attentional control processes, but not response conflict processes.

Individuals exhibited a greater flanker-related N2 amplitude interference effect while completing a high WM load task compared to a low WM load task (Qi et al., 2014; Wei and Zhou, 2020). An increased WM load reduces the available attentional control resources in order to perform the flanker task. We can interpret the increased N2 amplitude as a benchmark of response conflict (the first account). Hence, the increased WM load leads to fewer attentional control resources being available to inhibit distractors, and ultimately causes stronger N2 amplitude interference in incongruent trials. However, we interpreted the increased N2 amplitude as an indicator of top-down attentional control resource allocation (the second account). In the high WM load condition, although available attentional control resources are limited, individuals can exert greater effort to complete the flanker task; that is, they display increased recruitment of top-down attentional control, also displaying increased N2 amplitude. Both of the two explanations are logical and plausible.

Above all, the present study aimed to better understand how WM load influences flanker-related N2 amplitude, and then help us to better understand the underlying mechanism of the flanker-related N2 wave. In past studies, individuals were asked to perform an arrow flanker task while remembering either one digit (or letter, the low WM load) or six digits (or letters, the high WM load). For the current study, we used a more difficult WM task that further reduced the available attentional control resources during the flanker task. Individuals were asked to perform an arrow flanker task while remembering one letter (the low WM load), four letters (the middle WM load), or eight letters (the high WM load). In addition, a more difficult flanker task was used while the arrow was presented for 0.15 s instead of 0.3 s or longer as done in past studies (Qi et al., 2014; Wei and Zhou, 2020). By using these two methods, we predicted that individuals could complete the flanker task by exerting greater effort in the middle WM load, but could not do so by exerting greater effort in the high WM load, while the available attentional control resources were quite limited. Thus, if flanker-related N2 amplitude is an indicator of response conflict, increased WM load will increase flanker-related N2 amplitude, especially in incongruent trials. Conversely, if flanker-related N2 amplitude is an indicator of top-down attentional control resource allocation, the current middle WM load may increase flanker-related N2 amplitude, especially for incongruent trials. Notwithstanding, the currently high WM load will not increase flanker-related N2 amplitude, or even decrease flanker-related N2 amplitude.

In addition, in the flanker task, the ERP component P3 is a parietal stimulus-locked component with a latency between 300 and 600 ms (Polich, 2007). Flanker-related P3 amplitude is associated with task difficulty, which reveals the following pattern: it is smaller in experimental conditions providing incongruent response options than in experimental conditions providing congruent response options (Kok, 2001; Gonzalezvillar and Carrillodelapena, 2017). Concurrent WM tasks will decrease flanker-related P3 amplitude, especially in incongruent trials (Qi et al., 2014; Wei and Zhou, 2020). Hence, we hypothesized that an increased WM load would decrease flanker-related P3 amplitude, especially in incongruent trials.

## Materials and methods

### Participants

Desired sample size was based on G*Power analysis, we set the *f* = 0.25, α = 0.05, and Power = 0.9, G*Power produced a recommended sample size of 24 participants. In the present study, 40 right-handed participants were recruited from Suzhou University of Science and Technology in the People’s Republic of China (PRC). All participants gave written informed consent and were made aware of their right to withdraw at any time. The experimental procedures were approved by the Ethics Committee of Academic Committee in Suzhou University of Science and Technology and carried out in accordance with the approved guidelines. We excluded eight participants due to poor-quality electroencephalography (EEG) recordings with excessively large drifts. Thus, 32 participants (mean age = 19.88 ± 1.41 years, 17 females) were included in the final analysis.

### Stimuli

**FIGURE 1:**
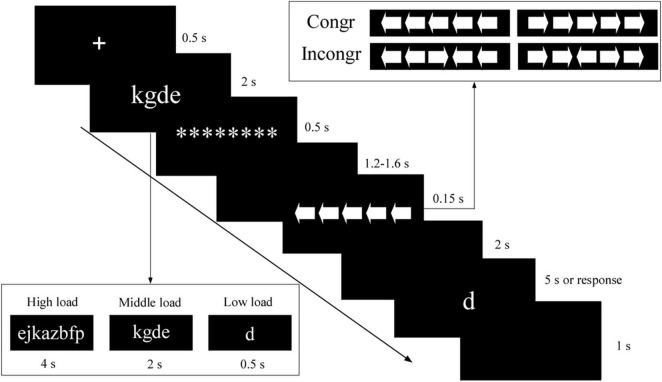
Task design. An example of a congruent trial in the middle WM load. Low load, low WM load; middle load, middle WM load; high load, high WM load; Incongr, incongruent trials; Congr, congruent trials; ********, a masking array with a row of eight asterisks.

We used a classical dual-task design (Wei and Zhou, 2020). As portrayed in Figure 1, in every experimental trial, the participants performed a WM task and an arrow flanker task simultaneously. First, a fixation cross was displayed for 0.5 s, followed by a low (presented for 0.5 s), middle (presented for 2 s), or high (presented for 4 s) “memory set” [i.e., a one-, four-, or eight- letter array (lowercase), with all letter(s) randomly drawn such that the letter array represented a meaningless “letter string”]. Then, a masking array with a row of eight asterisks was presented for 0.5 s. After a blank screen was shown for a randomized time between 1.2 and 1.5 s, the arrow flanker task was presented for 0.15 s, with 1 target arrow and 2 flanker arrows on each side. There were two conditions: (1) in the congruent condition (0.5 probability), the arrows were all facing the same direction; (2) in the incongruent condition (0.5 probability), the target and distractor arrows were facing opposite directions. The participants were asked to respond to the direction of the central arrow as quickly and accurately as possible by using their right hand to press “J” if the target arrow pointed to the left, or “K” if it pointed to the right. Each arrow in the string subtended a visual angle of 1.3° vertically and 1.3° horizontally. The distance between the arrows was 0.25°. Last, after a 2 s delay, a letter was presented. Using their left hand, the participants pressed “C” (yes) or “V” (no) to indicate whether the letter was present in the memory set. The memory probe was presented for 5 s or until the participant responded. The inter-trial interval was 1 s. All stimuli were white and appeared on a black background.

### Design and procedure

In a separate electromagnetic shielding room, the participants were seated comfortably about 70 cm away from a 17-inch LCD screen. The formal experiment consisted of three blocks, with 160 trials in each block. The participants were administered one of two sequences of experimental blocks (i.e., the first sequence: high/middle/low WM loads, and the second sequence: low/middle/high WM loads), which was counterbalanced across the participants so that half of them experienced the first sequence and the other half experienced the second sequence. In the flanker task, the participants were asked to complete the task accurately and quickly, but in the WM task, they were only asked to complete the task accurately. Before the formal experiment, they performed 24 practice trials (8 trials in the low, middle, and high WM load conditions).

### EEG data collection and analysis

We collected EEG data using 30 Ag-AgCl scalp electrodes (ANT Neuro system, 24-bit resolution) placed according to the international 10–20 system (pass-band: 0.01–100 Hz; sampling rate: 1000 Hz). While recording, the ground lead was located at the prefrontal lobe, and the CPz was set as a reference. Impedances were maintained below 10 kΩ.

We processed the EEG data using EEGLAB (Delorme and Makeig, 2004), an open-source toolbox running in a MATLAB environment. (1) Continuous EEG data were filtered with a 30 Hz low-pass filter and a 0.5 Hz high-pass filter, and then re-referenced to linked mastoids (i.e., using the average of both mastoids). (2) EEG epochs were extracted using a time window from −200 to 1000 ms, which was time-locked to the flanker stimulus onset; the epochs were baseline corrected using the pre-stimulus interval (−200 to 0 ms). (3) Trials with large drifts were manually removed, and trials contaminated by eyeblinks were corrected using an independent component analysis (ICA) algorithm (Delorme and Makeig, 2004). Across the participants, an average of 4.97 ± 1.53 ICAs of artifacts were identified as ocular artifacts by visual inspection, and were rejected. Only correct trials were included in the final analysis. (4) Finally, trials with amplitude values exceeding ±75 μV at any electrode were rejected. In the low load, the mean number of incongruent trials was 59.94 (SD = 16.73), the mean number of congruent trials was 61.38 (SD = 16.40); in the middle load, the mean number of incongruent trials was 59.19 (SD = 14.68), the mean number of congruent trials was 62.31 (SD = 13.40); in the high load, the mean number of incongruent trials was 49.22 (SD = 14.30), the mean number of congruent trials was 50.78 (SD = 10.98). In line with previous research (Kopp et al., 1996; Wei and Zhou, 2020) and based on visual inspection of ERP waveforms, the N2 amplitude was quantified as the mean amplitude of negative deflection at Cz between 230 and 380 ms after stimulus onset for incongruent and congruent trials separately, whereas the P3 amplitude was quantified as the mean amplitude of positive deflection at Pz between 350 and 600 ms after stimulus onset for incongruent and congruent trials separately.

### Statistical analysis

We carried out statistical analyses using IBM SPSS Statistics 23.0. To correct for the violation of the assumption of sphericity, we applied a Greenhouse–Geisser correction when necessary. We adjusted multiple comparisons using Bonferroni correction.

## Results

### Working memory task

We performed one-way repeated measures analysis of variance (ANOVA) on probe accuracy associated with the factor “WM load” (high, middle, and low). We found a significant main effect of WM load, *F*(1.37,42.46) = 108.54, *p* < 0.001, η^2^_*p*_ = 0.78. Follow-up analyses showed that the probe accuracy in the middle load (94.57 ± 0.85%) was significantly smaller than that in the low load (96.58 ± 0.45%, *p* = 0.04). The probe accuracy in the high load (78.91 ± 1.71%, *p* < 0.001) was significantly smaller than that in the middle load (*p* < 0.001) and low load (*p* < 0.001).

### Flanker task

#### Behavioral results

**FIGURE 2:**
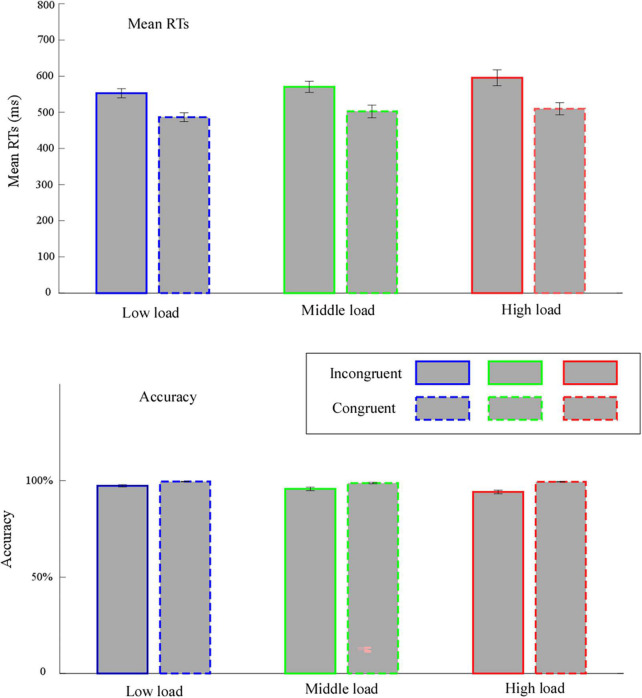
Mean values for reaction times (RTs) and accuracy. Error bars represent within-subject standard error of the means. Low load, low WM load; middle load, middle WM load; high load, high WM load.

The outcomes (mean values) of all dependent variables are presented in Figure 2.

#### Mean reaction times

We only considered RTs for correct trials in both the WM task and flanker task. We also discarded RTs exceeding ±3 SD of the individual mean scores.

We performed two-way repeated measures ANOVA on mean RTs associated with the factor’s “congruency” (congruent and incongruent) and “WM load” (high, middle, and low). We found a significant main effect of congruency, *F*(1,31) = 284.13, *p* < 0.001, η^2^_*p*_ = 0.90, implying that the mean RTs for incongruent trials (572.80 ± 15.91 ms) was significantly slower than for congruent trials (499.58 ± 14.95 ms). We witnessed a significant main effect of WM load, *F*(1.35,41.70) = 6.17, *p* = 0.01, η^2^_*p*_ = 0.17. Follow-up analyses showed that the mean RTs was significantly slower in the high load (552.58 ± 19.26 ms) than in the low load (519.54 ± 12.37 ms, *p* = 0.03). The interaction effect between congruency and WM load was significant, *F*(1.26,39.17) = 3.96, *p* = 0.045, η^2^_*p*_ = 0.11. Follow-up analyses revealed that: (1) the mean RTs for incongruent trials was significantly slower than for congruent trials in all WM loads (*p* < 0.001); (2) for incongruent trials, the mean RTs in the high load (595.44 ± 22.15 ms) was significantly slower than in the low load (552.62 ± 12.88 ms, *p* = 0.02); for congruent trials, the mean RTs have no significantly differences between each WM load (*p* ≥ 0.097).

We conducted one-way repeated measures ANOVA for the congruency effect with the WM load factor. We observed a significant main effect of WM load, *F*(1.26,39.17) = 3.96, *p* = 0.045, η^2^_*p*_ = 0.11. Follow-up analyses demonstrated that the congruency effect was significantly larger in the high load (85.73 ± 8.51 ms) than in the low load (66.16 ± 4.14 ms, *p* = 0.04).

#### Accuracy

We only analyzed accuracy when the probe trials were correct.

We performed a two-way repeated measures ANOVA for data accuracy. The outcomes revealed a significant main effect of congruency, *F*(1,31) = 41.45, *p* < 0.001, η^2^_*p*_ = 0.57, denoting that the accuracy for incongruent trials (95.74 ± 0.64%) was significantly lower than for congruent trials (99.58 ± 0.17%).

We observed a significant main effect of WM load, *F*(2,62) = 5.69, *p* = 0.005, η^2^_*p*_ = 0.16. Follow-up analyses showed that the accuracy in the high load (96.79 ± 0.54%) was significantly lower than in the low load (98.44 ± 0.31%, *p* = 0.02). The interaction effect between congruency and WM load was significant, *F*(2,62) = 6.05, *p* = 0.004, η^2^_*p*_ = 0.16. Follow-up analyses revealed that, (1) the accuracy for incongruent trials was significantly slower than for congruent trials in all WM loads (*p* ≤ 0.001); (2) for incongruent trials, the accuracy in the high load (94.17 ± 0.96%) was significantly lower than in the low load (97.33 ± 0.56%, *p* = 0.01); for congruent trials, the accuracy has no significantly differences between each WM load (*p* ≥ 0.119).

We conducted a one-way repeated measures ANOVA for the congruency effect with the WM load factor. The results uncovered a significant main effect of WM load, *F*(2,62) = 6.05, *p* = 0.004, η^2^_*p*_ = 0.16. Follow-up analyses implied that the congruency effect was significantly larger in the high load (−5.23 ± 0.87%) than in the low load (−2.21 ± 0.56%, *p* = 0.01) and in the middle load (−3.06 ± 0.79%, *p* = 0.04).

### Event-related potential

#### N2 amplitude

**FIGURE 3:**
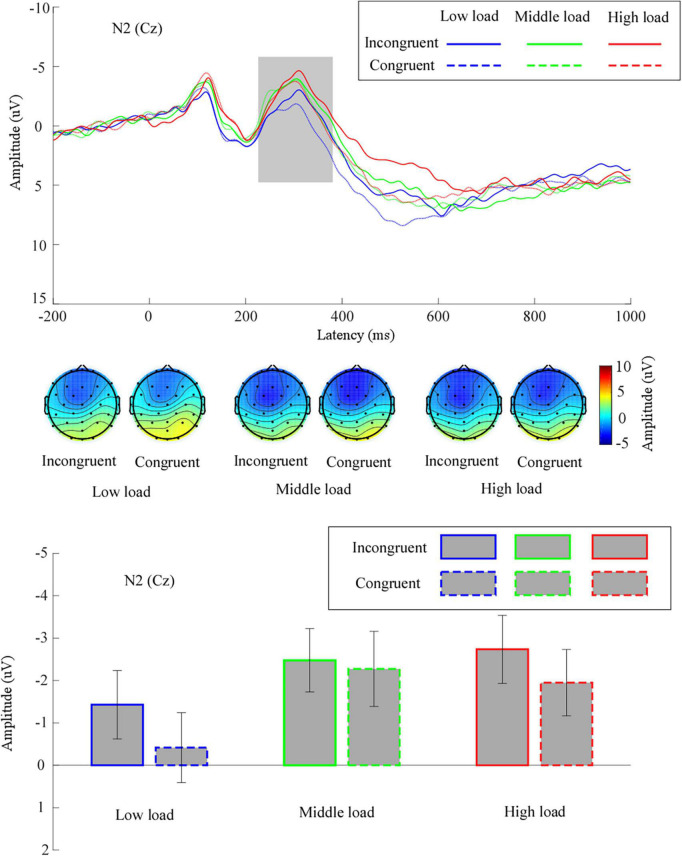
The grand means of N2’s waveforms across different conditions at electrode site Cz. Additionally, the topography of N2 (time window: 230–380 ms), and mean values for N2 amplitude are given. Low load, low WM load; middle load, middle WM load; high load, high WM load.

The grand means of the ERP waveforms and topographic scalp maps of N2 are presented in Figure 3.

We performed a two-way repeated measures ANOVA for the mean N2 amplitude. We noted a significant main effect of congruency, *F*(1,31) = 5.38, *p* = 0.03, η^2^_*p*_ = 0.15, suggesting that the mean N2 amplitude for incongruent trials (−2.21 ± 0.73 μV) was significantly larger than for congruent trials (−1.55 ± 0.75 μV). We observed a significant main effect of WM load, *F*(1.67,51.81) = 5.37, *p* = 0.01, η^2^_*p*_ = 0.15. Follow-up analyses revealed that the mean N2 amplitude in the middle load (−2.37 ± 0.79 μV) was significantly larger than in the low load (−0.92 ± 0.79 μV, *p* = 0.002). The mean N2 amplitude in the high load (−2.34 ± 0.77 μV) was marginally larger than in the low load (*p* = 0.06). There was no significant difference for the mean N2 amplitude between the middle load and the high load (*p* = 1). The interaction effect between congruency and WM load was not significant, *F*(2,62) = 1.24, *p* = 0.30, η^2^_*p*_ = 0.04.

#### P3 amplitude

**FIGURE 4:**
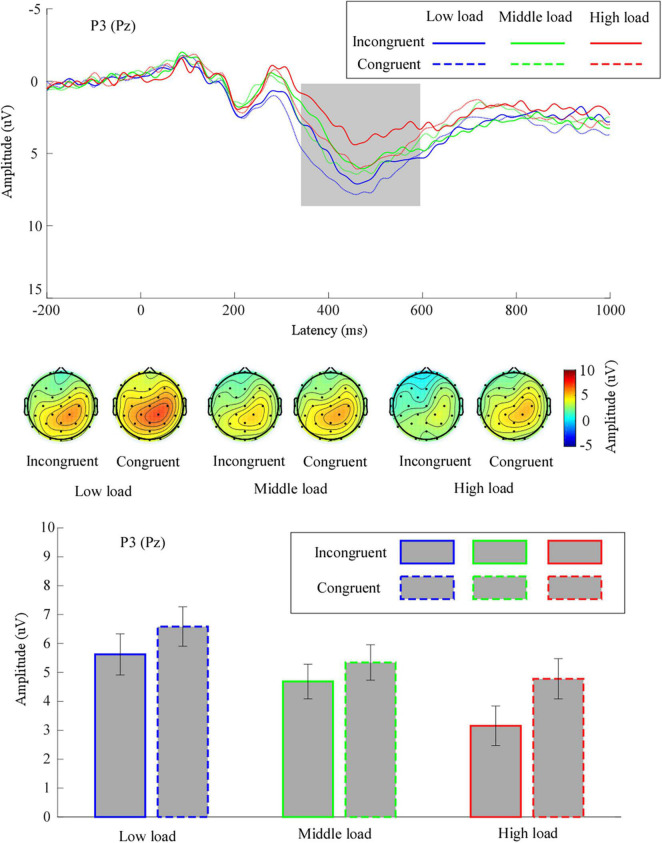
The grand means of P3’s waveforms across different conditions at electrode site Pz. Further, the topography of P3 (time window: 350–600 ms) and mean values for P3 amplitude are given. Low load, low WM load; middle load, middle WM load; high load, high WM load.

The grand means of the ERP waveforms and topographic scalp maps of P3 are depicted in Figure 4.

We performed a two-way repeated measures ANOVA on the mean P3 amplitude. The results pointed to a significant main effect of congruency, *F*(1,31) = 12.28, *p* = 0.001, η^2^_*p*_ = 0.28, suggesting that the mean P3 amplitude for incongruent trials (4.49 ± 0.60 μV) was significantly smaller than for the congruent trials (5.57 ± 0.60 μV). We noted a significant main effect of WM load, *F*(1.60,49.62) = 11.10, *p* < 0.001, η^2^_*p*_ = 0.26. Follow-up analyses demonstrated that the mean P3 amplitude in the middle load (5.02 ± 0.57 μV) was significantly smaller than in the low load (6.11 ± 0.67 μV, *p* = 0.04). The mean P3 amplitude in the high load (3.97 ± 0.66 μV) was significantly smaller than in the low load (*p* = 0.002) and in the middle load (*p* = 0.03). The interaction effect between congruency and WM load was not significant, *F*(2,62) = 2.57, *p* = 0.09, η^2^_*p*_ = 0.08.

## Discussion

Similarly to previous studies, the behavioral outcomes provide strong support for Lavie’s load theory of selective attention. The RTs and the accuracy results exhibited greater interference in the high WM load than in the low WM load. The N2 results offer support for the second account that the flanker-related N2 amplitude is an indicator of top-down attentional control resource allocation. Both the middle and high WM loads increased flanker-related N2 amplitude compared to the low WM load, but the high WM load did not increase flanker-related N2 amplitude compared to the middle WM load. In addition, in line with our hypothesis, the increased WM load decreased flanker-related P3 amplitude.

In good agreement with previous studies (Lavie et al., 2004; Lavie, 2005), the behavioral findings imply that increased WM load reduced the available attentional control resources to inhibit distractors, ultimately leading to a stronger interference effect. Because the RTs reflect the response conflict processes, we observed longer RTs, especially for incongruent trials, while increasing the WM load. In addition, the present study supports the idea that accuracy may also be associated with response conflict processes. We noted a lower incongruent accuracy with the high WM load than with the low WM load. This provides further evidence of Lavie’s load theory of selective attention (Lavie et al., 2004). In addition, the behavioral outcomes suggest that our experimental manipulation of these three WM loads was successful.

Importantly, the findings provide contrasting evidence for the first account of flanker-related N2, reflecting processes linked to response conflict. Increasing the WM load reduces the available attentional control resources to perform the flanker task (Lavie et al., 2004; Lavie, 2005). Using a more difficult flanker task, we found that individuals displayed a greater flanker-related N2 amplitude in the middle WM load than in the low WM load. If the flanker-related N2 reflects processes tied to response conflict, as in prior studies (Qi et al., 2014; Wei and Zhou, 2020), a greater flanker-related N2 amplitude interference effect should be seen while completing an additional WM load task. Moreover, we further reduced the available attentional control resources with a high WM load, which cannot increase flanker-related N2 amplitude. Thus, the findings support the second account of the flanker-related N2 reflecting processes linked to top-down attentional control resource allocation. In the middle WM load condition, although the available attentional control resources are limited, individuals can exert greater effort to complete the flanker task. Thus, they displayed increased recruitment of top-down attentional control, as shown by increased flanker-related N2 amplitude in the middle WM load (versus the low WM load). However, in the high WM load condition, available attentional control resources are further reduced; as such, individuals cannot exert greater effort to complete the flanker task. Hence, individuals exhibited no flanker-related N2 amplitude difference in the high WM load or the middle WM load.

The flanker task is a popular inhibition measurement tool in cognitive neuroscience (Larson et al., 2014). A different understanding of the flanker-related N2 outcomes may lead to a false conclusion (Groom and Cragg, 2015; Kałamała et al., 2018). Our present study offers fresh evidence for the idea that flanker-related N2 reflects top-down attentional control resource allocation (Tillman and Wiens, 2011). Compared to congruent trials, incongruent trials produce a larger N2 amplitude, which means that more attentional control processes are used to focus on the task-relevant aspects of a situation (Tillman and Wiens, 2011). This is a crucial reference value in related studies.

Similarly to previous studies, the P3 amplitude results provide evidence of Lavie’s load theory of selective attention (Lavie et al., 2004; Pratt et al., 2011; Wei and Zhou, 2020). P3 amplitude for incongruent trials was significantly smaller than for congruent trials, which added to supporting evidence that more difficult incongruent trials led to fewer attentional resources available for later inhibition processing. Increasing the WM load correspondingly reduces available attentional control resources and increases the difficulty of inhibiting irrelevant stimuli, culminating in decreased flanker-related P3 amplitude. However, in the work of Wei and Zhou (2020), an additional WM task reduced incongruent P3 amplitude. In the present study, additional WM tasks reduced both congruent and incongruent P3 amplitudes. The main reason for this discrepancy may be that a more difficult flanker task was employed in the present study, thereby making completion of the congruent trials more difficult while adding an additional WM task.

Our study has some limitations as well. The first limitation of the present study may explain why no flanker-related N2 amplitude difference between in the high WM load and the middle WM load. The participants may simplify the high WM load, and only focus on remembering a subset of the items, thus performing the task as if it was a middle WM load. Thus, we found no difference in flanker-related N2 amplitude between the two WM loads. Second, conflict adaptation effect analysis has beneficial transfer effects of explain which account is right. However, because the flanker trials were presented at random sequence and available flanker trials are quantity not sufficient, thus it is difficult and inappropriate to analysis the conflict adaptation effect in the present study. Further studies considering to avoid this limitation.

In sum, the present study provides new evidence of Lavie’s load theory of selective attention whereby completing an additional WM load reduced the availability of attentional control resources, in turn impairing inhibition processes during the flanker task. Further, the present study shows that individuals can exert greater effort to complete the flanker task, as depicted by a larger N2 amplitude while completing a middle or high WM load task. However, completing an additional high WM load task cannot increase flanker-related N2 amplitude (versus completing an additional middle WM load task). Thus, the findings support the account of the N2 processes being related to top-down attentional control resource allocation.

## Data availability statement

The original contributions presented in this study are included in the article/supplementary material, further inquiries can be directed to the corresponding author.

## Ethics statement

The studies involving human participants were reviewed and approved by the Academic Committee in Suzhou University of Science and Technology. Written informed consent to participate in this study was provided by the participants’ legal guardian/next of kin.

## Author contributions

HW, YY, and LZ conceived and designed the experiments and wrote the manuscript. HW and YY performed the experiments. HW analyzed the data. All authors contributed to the article and approved the submitted version.

## References

[B1] Bartholow B. D. Pearson M. A. Dickter C. L. Sher K. J. Fabiani M. Gratton G. (2005). Strategic control and medial frontal negativity: Beyond errors and response conflict. *Psychophysiology* 42 33–42. 10.1111/j.1469-8986.2005.00258.x 15720579

[B2] Carter C. S. Braver T. S. Barch D. M. Botvinick M. M. Noll D. Cohen J. D. (1998). Anterior cingulate cortex, error detection, and the online monitoring of performance. *Science* 280 747–749. 10.1126/science.280.5364.747 9563953

[B3] Clayson P. E. Larson M. J. (2011). Conflict adaptation and sequential trial effects: Support for the conflict monitoring theory. *Neuropsychologia* 49 1953–1961. 10.1016/j.neuropsychologia.2011.03.023 21435347

[B4] Delorme A. Makeig S. (2004). EEGLAB: An open source toolbox for analysis of single-trial EEG dynamics including independent component analysis. *J. Neurosci. Methods* 134 9–21. 10.1016/j.jneumeth.2003.10.009 15102499

[B5] Folstein J. R. Van Petten C. (2008). Influence of cognitive control and mismatch on the N2 component of the ERP: A review. *Psychophysiology* 45 152–170. 10.1111/j.1469-8986.2007.00602.x 17850238 PMC2365910

[B6] Gonzalezvillar A. J. Carrillodelapena M. T. (2017). Brain electrical activity signatures during performance of the multisource interference task. *Psychophysiology* 54 874–881. 10.1111/psyp.12843 28220517

[B7] Groom M. J. Cragg L. (2015). Differential modulation of the N2 and P3 event-related potentials by response conflict and inhibition. *Brain Cogn.* 97 1–9. 10.1016/j.bandc.2015.04.004 25955278

[B8] Kałamała P. Szewczyk J. Senderecka M. Wodniecka Z. (2018). Flanker task with equiprobable congruent and incongruent conditions does not elicit the conflict N2. *Psychophysiology* 55:e12980. 10.1111/psyp.12980 28845513

[B9] Kok A. (2001). On the utility of P3 amplitude as a measure of processing capacity. *Psychophysiology* 38 557–577. 10.1017/S0048577201990559 11352145

[B10] Kopp B. Rist F. Mattler U. (1996). N200 in the flanker task as a neurobehavioral tool for investigating executive control. *Psychophysiology* 33 282–294. 10.1111/j.1469-8986.1996.tb00425.x 8936397

[B11] Larson M. J. Clayson P. E. Ann C. (2014). Making sense of all the conflict: A theoretical review and critique of conflict-related ERPs. *Int. J. Psychophysiol.* 93 283–297. 10.1016/j.ijpsycho.2014.06.007 24950132

[B12] Lavie N. (2005). Distracted and confused?: Selective attention under load. *Trends Cogn. Sci.* 9 75–82. 10.1016/j.tics.2004.12.004 15668100

[B13] Lavie N. (2010). Attention, distraction, and cognitive control under load. *Curr. Dir. Psychol. Sci.* 19 143–148. 10.1177/0963721410370295 26728138

[B14] Lavie N. Hirst A. De Fockert J. W. Viding E. (2004). Load theory of selective attention and cognitive control. *J. Exp. Psychol. Gen.* 133 339–354. 10.1037/0096-3445.133.3.339 15355143

[B15] Murphy G. Groeger J. A. Greene C. M. (2016). Twenty years of load theory—Where are we now, and where should we go next? *Psychon. Bull. Rev.* 23 1316–1340. 10.3758/s13423-015-0982-5 26728138

[B16] Polich J. (2007). Updating P300: An integrative theory of P3a and P3b. *Clin. Neurophysiol.* 118 2128–2148. 10.1016/j.clinph.2007.04.019 17573239 PMC2715154

[B17] Pratt N. Willoughby A. Swick D. (2011). Effects of working memory load on visual selective attention: Behavioral and electrophysiological evidence. *Front. Hum. Neurosci.* 5:57. 10.3389/fnhum.2011.00057 21716633 PMC3115462

[B18] Qi S. Zeng Q. Luo Y. Duan H. Ding C. Hu W. (2014). Impact of working memory load on cognitive control in trait anxiety: An ERP study. *PLoS One* 9:e111791. 10.1371/journal.pone.0111791 25369121 PMC4219777

[B19] Tillman C. M. Wiens S. (2011). Behavioral and ERP indices of response conflict in Stroop and Flanker tasks. *Psychophysiology* 48 1405–1411. 10.1111/j.1469-8986.2011.01203.x 21457276

[B20] Van Veen V. Carter C. S. (2002a). The anterior cingulate as a conflict monitor: FMRI and ERP studies. *Physiol. Behav.* 77 477–482. 10.1016/S0031-9384(02)00930-7 12526986

[B21] Van Veen V. Carter C. S. (2002b). The timing of action-monitoring processes in the anterior cingulate cortex. *J. Cogn. Neurosci.* 14 593–602. 10.1162/08989290260045837 12126500

[B22] Wei H. De Beuckelaer A. Zhou R. (2021). Enhanced or impoverished recruitment of top-down attentional control of inhibition in test anxiety. *Biol. Psychol.* 161:108070. 10.1016/j.biopsycho.2021.108070 33722566

[B23] Wei H. Zhou R. (2020). High working memory load impairs selective attention: EEG signatures. *Psychophysiology* 57:e13643. 10.1111/psyp.13643 32725929

[B24] Yeung N. Botvinick M. M. Cohen J. D. (2004). The neural basis of error detection: Conflict monitoring and the error-related negativity. *Psychol. Rev.* 111 931–959. 10.1037/0033-295X.111.4.939 15482068

[B25] Yeung N. Nieuwenhuis S. (2009). Dissociating response conflict and error likelihood in anterior cingulate cortex. *J. Neurosci.* 29 14506–14510. 10.1523/JNEUROSCI.3615-09.2009 19923284 PMC2831178

[B26] Zhang W. Luck S. (2015). Opposite effects of capacity load and resolution load on distractor processing. *J. Exp. Psychol. Hum. Percept. Perform.* 41 22–27. 10.1037/xhp0000013 25365573 PMC4308516

